# Synthesis and characterisation of Ba(Zn_1−*x*_Co_*x*_)_2_Si_2_O_7_ (0 ≤ *x* ≤ 0.50) for blue-violet inorganic pigments

**DOI:** 10.1039/c8ra00101d

**Published:** 2018-02-27

**Authors:** Takashi Tsukimori, Yusuke Shobu, Ryohei Oka, Toshiyuki Masui

**Affiliations:** Department of Chemistry and Biotechnology, Graduate School of Engineering, Tottori University 4-101, Koyama-cho Minami Tottori 680-8552 Japan masui@chem.tottori-u.ac.jp +81-857-31-5264 +81-857-31-5264

## Abstract

Ba(Zn_1−*x*_Co_*x*_)_2_Si_2_O_7_ (0 ≤ *x* ≤ 0.50) solid solutions were synthesized as novel blue-violet inorganic pigments by a conventional solid-state reaction method. The crystal structure, optical properties, and colour of the pigments were characterized. All the pigments were obtained in a single-phase form. The pigments strongly absorbed visible light at wavelengths from 550 to 650 nm, corresponding to the range of green to orange light. This optical absorption was caused by the d–d transition of the tetrahedrally coordinated Co^2+^ (^4^A_2_(F) → ^4^T_1_(P)), which was the origin of the blue-violet colour of the pigments. The most intense colour was obtained for Ba(Zn_0.85_Co_0.15_)_2_Si_2_O_7_, where a* = +52.2 and b* = −65.5 in the CIE (Commission Internationale de l'Éclairage) L*a*b* system. These absolute values were significantly larger than those of commercial violet pigments such as Co_3_(PO_4_)_2_ (a* = +33 and b* = −32) and NH_4_MnP_2_O_7_ (a* = +39 and b* = −21). Therefore, the Ba(Zn_0.85_Co_0.15_)_2_Si_2_O_7_ pigment could be a novel blue-violet inorganic pigment.

## Abstract 2

Ba(Zn_1−*x*_Co_*x*_)_2_Si_2_O_7_ (0 ≤ *x* ≤ 0.50) solid solutions were synthesized as blue-violet inorganic pigments and the colour gradually changed from pale blue-violet to deep blue-violet with increasing the Co^2+^ concentration.

## Graphical abstract

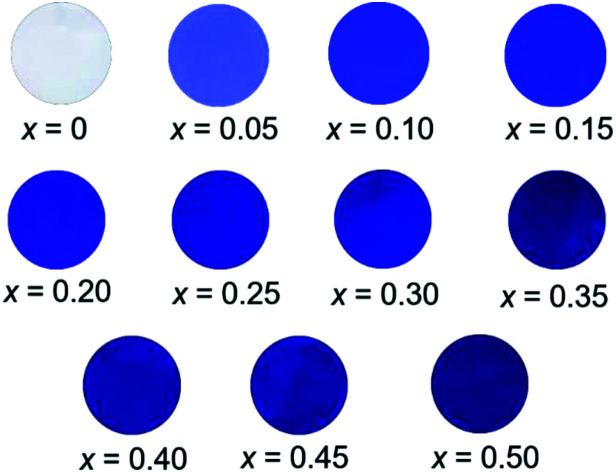

## Introduction

Cobalt (Co) and its compounds have been applied in alloys, paints, catalysts, cements and ceramic pigments.^1–5^ In particular, the Co^2+^ ion is often employed as a colouring source for blue and violet pigments. There are a number of reports on blue pigments using Co^2+^ ions, such as Co_2_SiO_4_ olivine,^6^ (Co, Zn)_2_SiO_4_ willemite,^7^ CoAl_2_O_4_ spinel^8^ and Co_2_SnO_4_.^9^ The colouring performance of these pigments mainly depends on the coordination number around the Co^2+^ ion, which is very important for the appearance of the bluish colour. However, the use of a large amount of Co increases the cost of the material, which becomes expensive because of its rarity.

Accordingly, selection of an appropriate host lattice, minimization of the Co content and strong colouring performance are necessary, when Co is applied to colour pigments. Although many studies on the reduction of the amount of Co in the pigments have been reported,^10–14^ almost of them are on the blue pigments and there are only a few reports on the violet pigments.^13,15,16^

Generally, the colour of inorganic pigments is mainly affected by the crystal field, generated by the ions surrounding the chromophore.^17^ Cobalt violet (Co_3_(PO_4_)_2_) and manganese violet (NH_4_MnP_2_O_7_) has been well known as current commercial violet pigments. The structure of Co_3_(PO_4_)_2_ is formed by distorted trigonal bipyramids CoO_5_, fairly regular CoO_6_ octahedra and almost regular PO_4_ tetrahedra.^13^ Generally, cobalt ion can take various coordination numbers and represent various colours in phosphates, In Co_3_(PO_4_)_2_·8H_2_O, Co^2+^ has octahedral 6 coordination to show reddish violet colour, while in KCoPO_4_ it has tetrahedral 4 coordination to evince blue-violet colour. On the other hand, NH_4_MnP_2_O_7_ presents violet colour due to the d–d transition of the octahedral coordinated Mn^3+^.^18^ However, the vividness of the Co_3_(PO_4_)_2_ and the NH_4_MnP_2_O_7_ pigments is insufficient, that is, their absolute values of a* and b* in the CIE L*a*b* system are not so large. In this system, the parameter L* indicate the brightness or darkness of a colour on relation to a neutral grey scale, while the parameters a* (the red-green axis) and b* (the yellow-blue axis) express the colour qualitatively. In addition, the thermal resistance of NH_4_MnP_2_O_7_ is not enough, because it is decomposed around 340 °C.^18^ Some violet pigments without Co have been proposed recently,^19–21^ but their colours are not much different from those of the existing commercial violet pigments. Organic pigments are inferior to inorganic pigments in heat resistance and weather resistance. Thus, it is significant to synthesize a novel violet inorganic pigment in which the colour property is improved.

Because of this situation, we focused on barium zinc silicate BaZn_2_Si_2_O_7_ as a host lattice of the novel violet pigment. This compound has a layered structure composed of [ZnO_4_]^2−^ tetrahedra connected at each corner to [SiO_4_] tetrahedra. Each [SiO_4_] tetrahedron is connected over three corners to one [SiO_4_] and two [ZnO_4_]^2−^ tetrahedra, and the forth corner is a non-bridging oxygen atom. The Ba^2+^ ions are located in between the zinc silicate layers.^22,23^ As a related compound, Ba(M, Ni)_2_Si_2_O_7_ (M = Zn or Mg) pigments have been ever reported, but they exhibit red and purplish red colours due to the d–d transition of the tetrahedral coordinated Ni^2+^.^24^ In this study, Ba(Zn_1−*x*_Co_*x*_)_2_Si_2_O_7_ (0 ≤ *x* ≤ 0.50) pigments were synthesized by a conventional solid-state reaction method, and the colour properties of the pigments were investigated as novel blue-violet inorganic pigments.

## Experimental

### Materials and methods

The Ba(Zn_1−*x*_Co_*x*_)_2_Si_2_O_7_ (0 ≤ *x* ≤ 0.50) pigments were synthesized by a conventional solid-state reaction method. BaCO_3_ (Kishida Chemical, Japan), ZnO (Kishida Chemical, Japan), SiO_2_ (Wako Pure Chemical, Japan) and Co_3_O_4_ (Wako Pure Chemical, Japan) were used as starting materials. The raw materials were mixed in a stoichiometric amount in an agate mortar. The mixture was calcined in an alumina boat at 1250 °C for 6 h. The samples were ground in an agate mortar before characterisation.

### Characterisation

The composition of the samples was confirmed by X-ray fluorescence spectroscopy (XRF; Rigaku, ZSX Primus). The crystal structures of the samples were identified by X-ray powder diffraction (XRD; Rigaku, Ultima IV) with Cu-Kα radiation (40 kV, 40 mA). The sampling width and the scan speed were 0.02° and 6.0 min^−1^, respectively. The lattice parameters and volumes were calculated from the XRD peak angles, which were refined using α-Al_2_O_3_ as a standard and using the CellCalc Ver. 2.20 software. The size and morphology of the Ba(Zn_0.85_Co_0.15_)_2_Si_2_O_7_ particles were observed by using scanning electron microscopy (SEM; JEOL, JSM-6701F). Gold was sputtered before observation to avoid the charge-up of the samples. The purity of the samples was analysed by energy dispersive X-ray analysis (EDX; Oxford Instruments, INCA Energy). An X-ray photoelectron spectrum (XPS; ULVAC-PHI, PHI5000 VersaProve II) of the Ba(Zn_0.85_Co_0.15_)_2_Si_2_O_7_ pigment was measured using Mg-Kα radiation to investigate the oxidation state of the Co ion.

The optical reflectance of the Ba(Zn_1−*x*_Co_*x*_)_2_Si_2_O_7_ (0 ≤ *x* ≤ 0.50) samples were measured using a UV-Vis spectrometer (Shimadzu, UV-2550) with barium sulphate as a reference. The colour properties of the samples were estimated in terms of the CIE L*a*b*Ch° system using a calorimeter (Konica-Minolta, CR-300). This colour measurement was made for powder samples. In the case of the blue-violet pigment, positive a* and negative b* values are desirable. Chroma parameter (*C*) represents the colour saturation of the pigments and is calculated according to the following formula: *C* = [(a*)^2^ + (b*)^2^]^1/2^. The parameter *h*° ranges from 0 to 360° (300 ≤ *h*° ≤ 330 means blue-violet), and is calculated with the formula, *h*° = tan^−1^(b*/a*).

## Results and discussion

### X-ray fluorescence analysis (XRF)

The XRF analysis data of the samples were listed in Table 1. All compositions were almost in good agreement with those of the nominal stoichiometric ones.

**Table 1:** The compositions of the Ba(Zn_1−*x*_Co_*x*_)_2_Si_2_O_7_ (0 ≤ *x* ≤ 0.50) pigments

Stoichiometric composition	Analysed composition
BaZn_2_Si_2_O_7_	Ba_0.99_Zn_1.99_Si_2.02_O_7.02_
Ba(Zn_0.95_Co_0.05_)_2_Si_2_O_7_	Ba_0.95_(Zn_0.92_Co_0.04_)_2_Si_2.14_O_7.15_
Ba(Zn_0.90_Co_0.10_)_2_Si_2_O_7_	Ba_1.04_(Zn_0.87_Co_0.11_)_2_Si_2.00_O_7.00_
Ba(Zn_0.85_Co_0.15_)_2_Si_2_O_7_	Ba_1.05_(Zn_0.84_Co_0.15_)_2_Si_1.96_O_6.96_
Ba(Zn_0.80_Co_0.20_)_2_Si_2_O_7_	Ba_0.94_(Zn_0.81_Co_0.22_)_2_Si_2.00_O_7.00_
Ba(Zn_0.75_Co_0.25_)_2_Si_2_O_7_	Ba_1.00_(Zn_0.76_Co_0.24_)_2_Si_2.00_O_7.00_
Ba(Zn_0.70_Co_0.30_)_2_Si_2_O_7_	Ba_1.05_(Zn_0.71_Co_0.29_)_2_Si_2.14_O_7.14_
Ba(Zn_0.65_Co_0.35_)_2_Si_2_O_7_	Ba_0.97_(Zn_0.63_Co_0.34_)_2_Si_2.10_O_7.10_
Ba(Zn_0.60_Co_0.40_)_2_Si_2_O_7_	Ba_0.95_(Zn_0.58_Co_0.37_)_2_Si_2.15_O_7.15_
Ba(Zn_0.55_Co_0.45_)_2_Si_2_O_7_	Ba_1.03_(Zn_0.54_Co_0.42_)_2_Si_2.05_O_7.05_
Ba(Zn_0.50_Co_0.50_)_2_Si_2_O_7_	Ba_1.00_(Zn_0.51_Co_0.47_)_2_Si_2.05_O_7.05_

### X-ray powder diffraction (XRD)

**Fig. 1:**
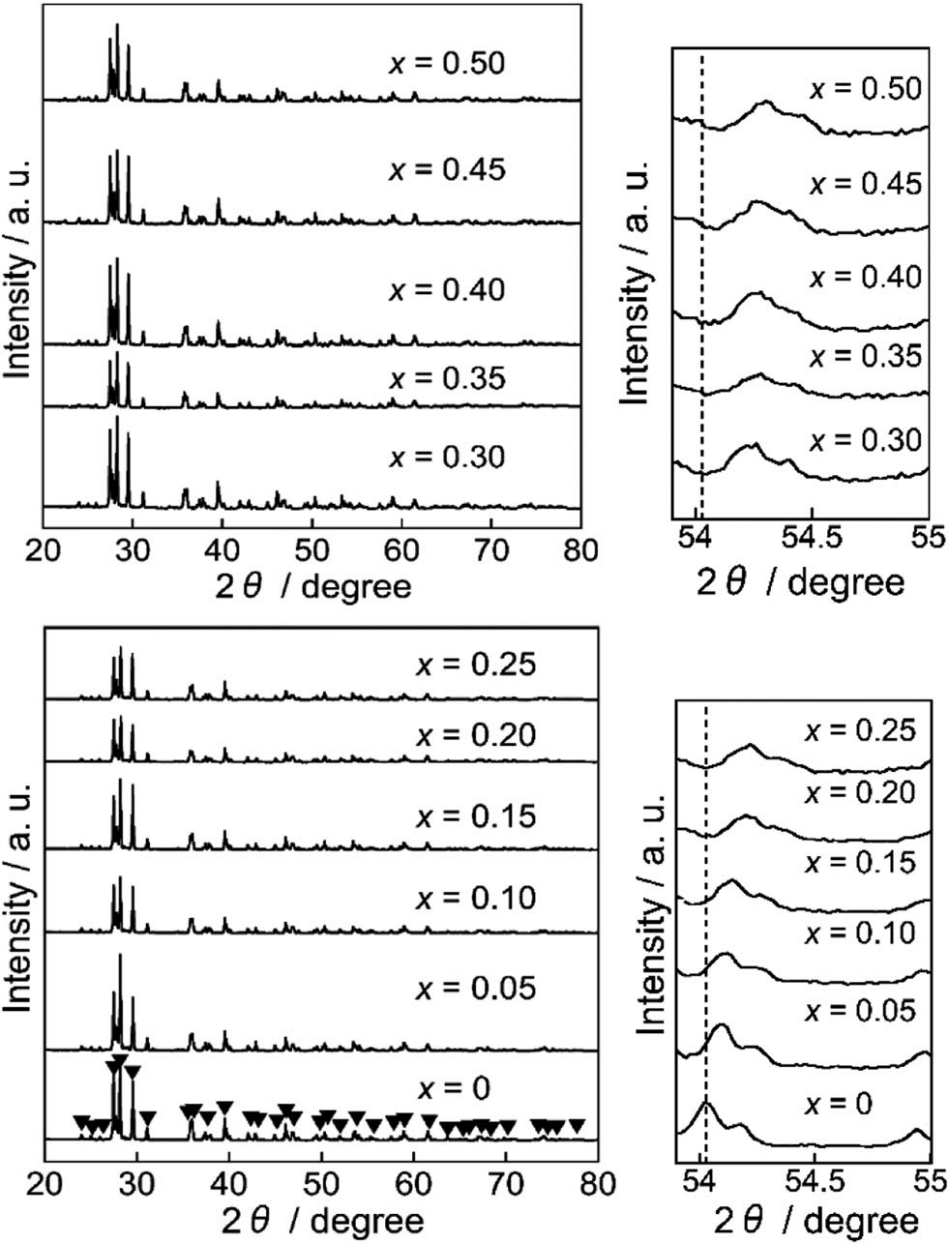
XRD patterns of the Ba(Zn_1−*x*_Co_*x*_)_2_Si_2_O_7_ (0 ≤ *x* ≤ 0.50) pigments.

Fig. 1 shows the XRD patterns of the Ba(Zn_1−*x*_Co_*x*_)_2_Si_2_O_7_ (0 ≤ *x* ≤ 0.50) pigments. All samples were obtained in a single-phase form and the diffraction patterns were well indexed to that of the monoclinic BaZn_2_Si_2_O_7_ structure whose space group was *C*2/*c*.^22,23,25^ This structure is different from that of orthorhombic BaCu_2_Si_2_O_7_ (ref. 26) having a similar composition. The diffraction peaks about 54° shifted to higher angles with increasing the Co^2+^ content. The lattice volumes of the Ba(Zn_1−*x*_Co_*x*_)_2_Si_2_O_7_ (0 ≤ *x* ≤ 0.50) samples calculated from the diffraction peaks are summarized in Table 2. The volume decreased with increasing the Co^2+^ concentration, indicating that the Zn^2+^ (ionic radius: 0.060 nm)^27^ ions were partially substituted with the smaller Co^2+^ (ionic radius: 0.058 nm)^27^ ions and the solid solutions based on monoclinic BaZn_2_Si_2_O_7_ were successfully synthesized in a single-phase form.

**Table 2:** Lattice volumes of the Ba(Zn_1−*x*_Co_*x*_)_2_Si_2_O_7_ (0 ≤ *x* ≤ 0.50) pigments

*x*	Lattice volume/nm^3^
0	1.27603
0.05	1.27570
0.10	1.27559
0.15	1.27555
0.20	1.27497
0.25	1.27463
0.30	1.27449
0.35	1.27383
0.40	1.27370
0.45	1.27356
0.50	1.27330

### Scanning electron microscopic (SEM) image and energy dispersive X-ray (EDX) analysis

**Fig. 2:**
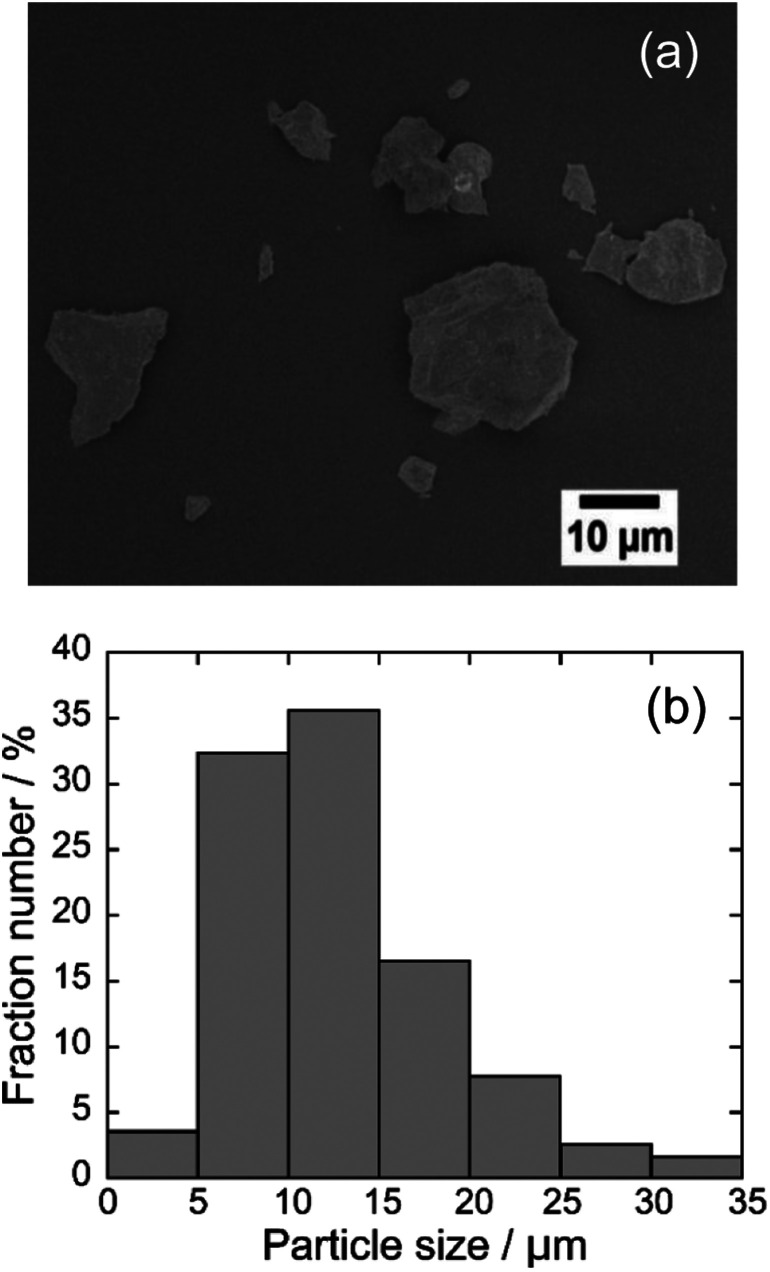
SEM image (a) and particle distribution (b) of the Ba(Zn_0.85_Co_0.15_)_2_Si_2_O_7_ pigment.

**Fig. 3:**
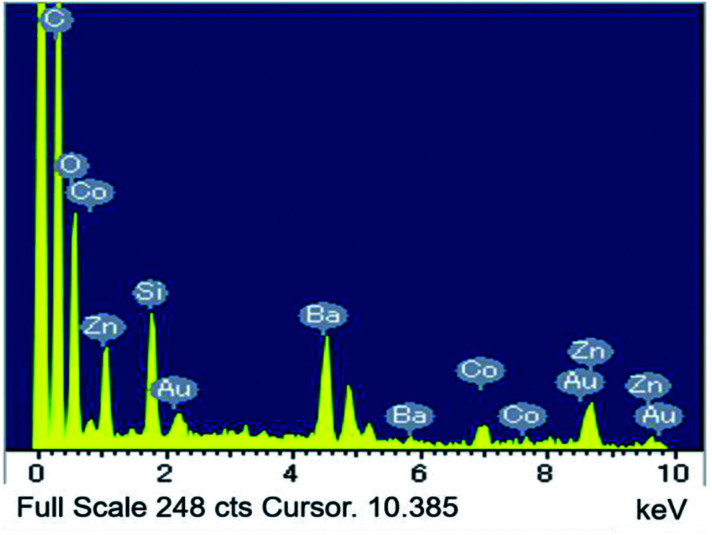
The EDS analysis for Ba(Zn_0.85_Co_0.15_)_2_Si_2_O_7_.

**Fig. 4:**
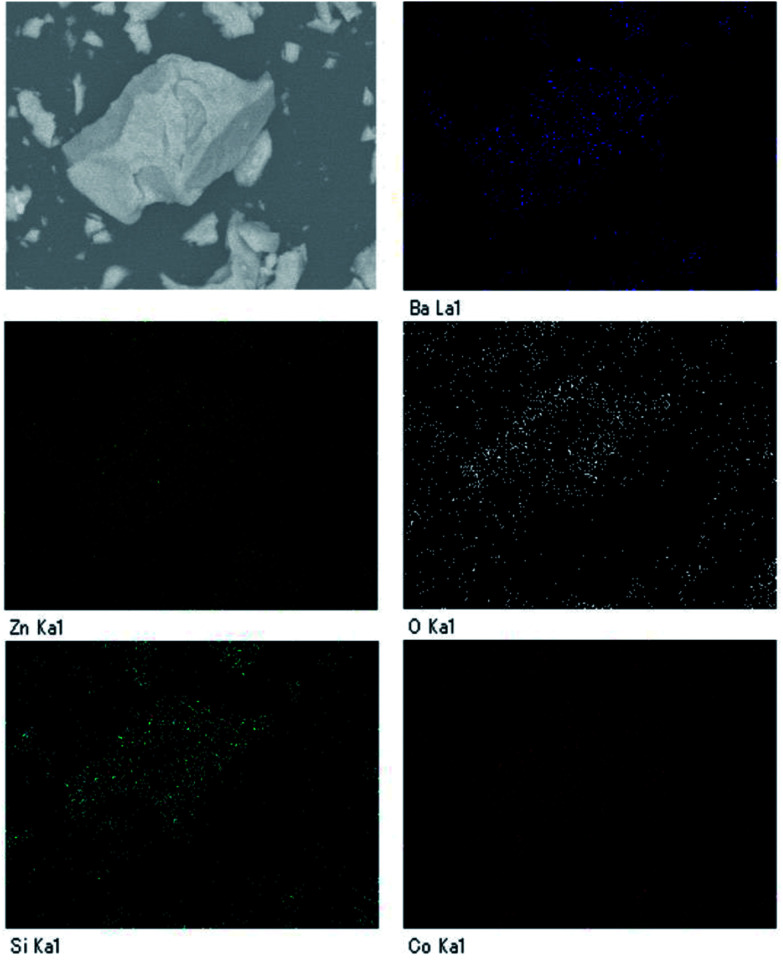
The X-ray dot mapping analysis of the Ba(Zn_0.85_Co_0.15_)_2_Si_2_O_7_.

Fig. 2 depicts the SEM image and particle distribution of the Ba(Zn_0.85_Co_0.15_)_2_Si_2_O_7_ pigment. The average particle size calculated from 400 particles was about 13 μm. The EDX analysis result for the Ba(Zn_0.85_Co_0.15_)_2_Si_2_O_7_ sample is shown in Fig. 3. It was confirmed that Ba, Zn, Si, Co and O were present and non-impurities were observed without Au (charge-up preventer). The X-ray dot mapping analysis results is depicted in Fig. 4, indicating that the component elements were uniformly distributed in the particle.

### X-ray photoelectron spectrum (XPS)

**Fig. 5:**
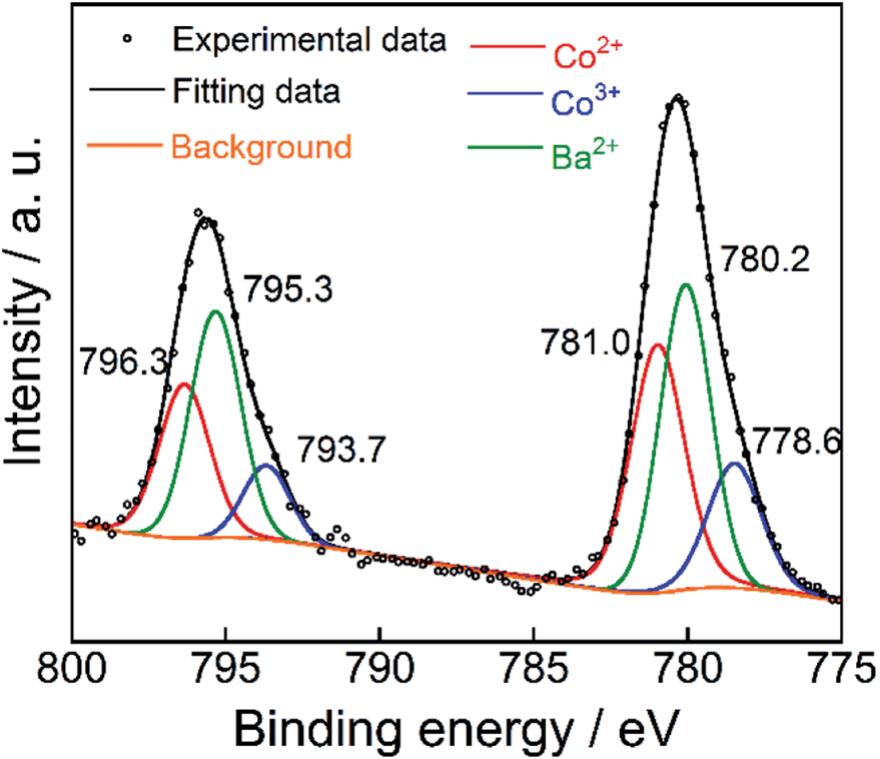
XPS of Co 2p and Ba 3d on the surface of the Ba(Zn_0.85_Co_0.15_)_2_Si_2_O_7_ pigment.

The XPS of the Ba(Zn_0.85_Co_0.15_)_2_Si_2_O_7_ pigment is shown in Fig. 5. This spectrum was deconvoluted into three components, considering the spin–orbit doublets. The intense peaks at 795.3 eV and 780.2 eV were attributed to the Ba^2+^ 3d_3/2_ and 3d_5/2_ configurations, respectively.^28,29^ Although the small peaks observed at 793.7 eV and 778.6 eV were assigned to the Co^3+^ 2p_3/2_ and 2p_1/2_ lines, more intense peaks were also detected at 796.3 eV and 781.0 eV, corresponding to those of Co^2+^.^29–31^ These results indicate that the dominant oxidation state of cobalt ions was divalent on the surface of the Ba(Zn_0.85_Co_0.15_)_2_Si_2_O_7_ pigment. Furthermore, the d–d transition of the tetrahedral coordinated Co^3+^ ions was appeared around 9000 cm^−1^ (1111 nm).^32^ Therefore, the Co^3+^ ions do not affect the colour of the present Ba(Zn_1−*x*_Co_*x*_)_2_Si_2_O_7_ (0.05 ≤ *x* ≤ 0.50) pigments.

### Reflectance spectra

**Fig. 6:**
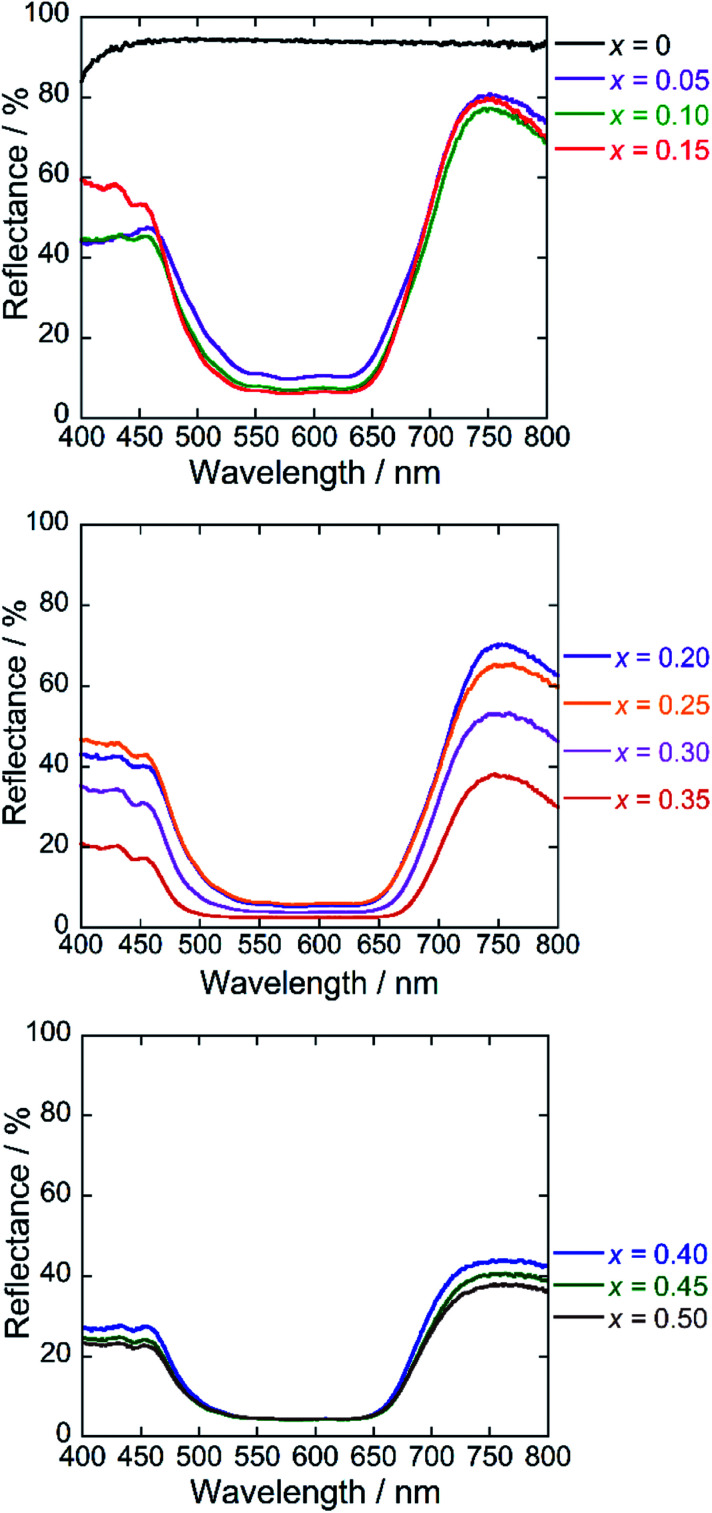
UV-Vis reflectance spectra of the Ba(Zn_1−*x*_Co_*x*_)_2_Si_2_O_7_ (0 ≤ *x* ≤ 0.50) pigments.

Fig. 6 depicts the UV-Vis diffuse reflectance spectra for the Ba(Zn_1−*x*_Co_*x*_)_2_Si_2_O_7_ (0 ≤ *x* ≤ 0.50) pigments. High reflectance was observed in the visible light region for the Co^2+^-free BaZn_2_Si_2_O_7_ (*x* = 0) sample. On the other hand, strong absorption bands originated by the d–d transition of tetrahedral coordinated Co^2+^ (ref. 8, 33 and 34) were observed in the Ba(Zn_1−*x*_Co_*x*_)_2_Si_2_O_7_ (0.05 ≤ *x* ≤ 0.50) pigments from 550 to 650 nm corresponding to the green-orange lights. The Co^2+^ ion has the d^7^ electron configuration and the energy level structure of the Co^2+^ ion in a tetrahedral site is similar to that of d^3^ ion in an octahedral site.^35^ According to the Tanabe–Sugano diagram, the bands from 550 to 650 nm are assigned to the ^4^A_2_(F) → ^4^T_1_(P) transition of the tetrahedral coordinated Co^2+^.^33–36^

### Chromatic properties

**Fig. 7:**
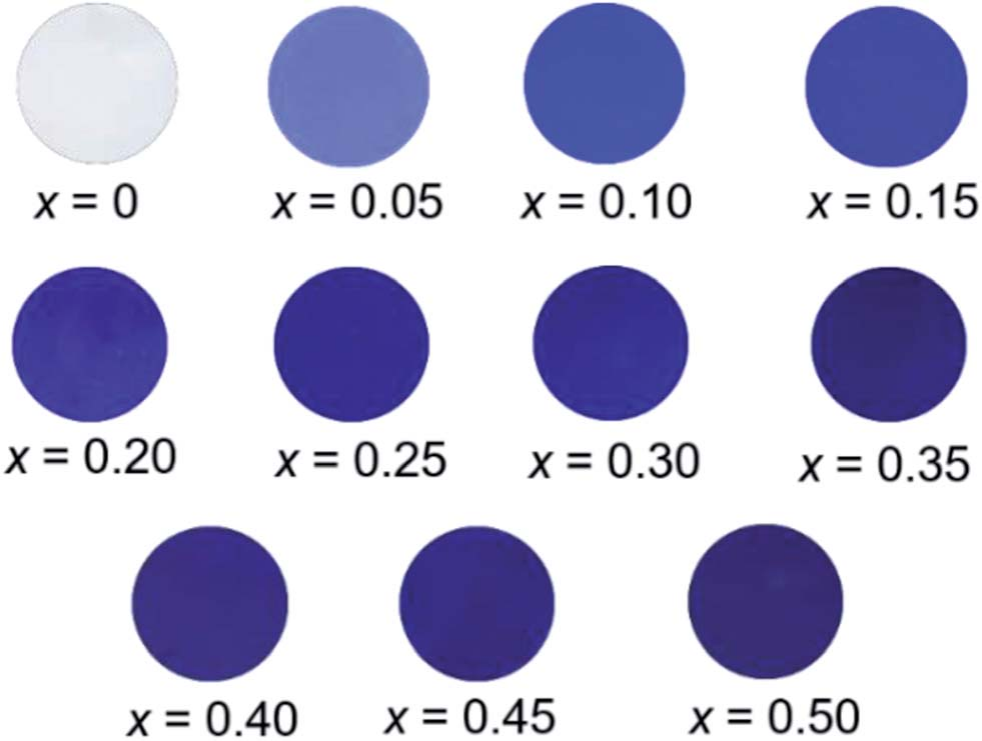
Photographs of the Ba(Zn_1−*x*_Co_*x*_)_2_Si_2_O_7_ (0 ≤ *x* ≤ 0.50) pigments.

The L*a*b*Ch° colour coordinate data for the Ba(Zn_1−*x*_Co_*x*_)_2_Si_2_O_7_ (0 ≤ *x* ≤ 0.50) pigments are summarized in Table 3. They were compared using powder samples. It is obvious that the a* and b* values became significantly positive and negative, respectively, by the introduction of Co^2+^ in the host BaZn_2_Si_2_O_7_ lattice. As mentioned in the previous section, the Ba(Zn_1−*x*_Co_*x*_)_2_Si_2_O_7_ pigments absorbed the green-orange lights but reflected the complementary blue and red lights. This is the reason for that positive a* and negative b* values were obtained in these pigments. The photographs of the Ba(Zn_1−*x*_Co_*x*_)_2_Si_2_O_7_ (0 ≤ *x* ≤ 0.50) samples are shown in Fig. 7. The colour of the Ba(Zn_1−*x*_Co_*x*_)_2_Si_2_O_7_ (0 ≤ *x* ≤ 0.50) pigments gradually changed from white to dark blue-violet as the Co^2+^ concentration increased. Among the samples synthesized in this study, the largest absolute values in the colour coordinate data were obtained for Ba(Zn_0.85_Co_0.15_)_2_Si_2_O_7_ (a* = +52.2 and b* = −65.5).

**Table 3:** The L*a*b*Ch° colour coordinates of the Ba(Zn_1−*x*_Co_*x*_)_2_Si_2_O_7_ (0 ≤ *x* ≤ 0.50) pigments

*x*	L*	a*	b*	*C*	*h*°
0	93.7	−0.01	+0.20	0.20	92.9
0.05	43.8	+27.9	−46.6	54.3	300.9
0.10	30.1	+39.8	−54.8	67.7	306.0
0.15	28.6	+52.2	−65.5	83.8	308.6
0.20	25.9	+44.9	−57.8	76.4	307.8
0.25	21.8	+49.9	−59.8	77.9	309.8
0.30	25.0	+33.3	−45.4	56.3	306.3
0.35	22.8	+27.4	−38.0	46.8	305.8
0.40	21.0	+35.9	−45.9	58.3	308.0
0.45	20.2	+33.4	−43.3	54.6	307.6
0.50	20.8	+30.4	−40.5	50.6	306.9

They were compared with those of the commercially available Co_3_(PO_4_)_2_ and NH_4_MnP_2_O_7_ pigments in Table 4. It is notable that the absolute values of a* and b* for the Ba(Zn_0.85_Co_0.15_)_2_Si_2_O_7_ were significantly larger than those for the commercial violet pigments.

**Table 4:** The L*a*b*Ch° colour coordinates of the Ba(Zn_0.85_Co_0.15_)_2_Si_2_O_7_ pigment and commercial violet pigments

Samples	L*	a*	b*	*C*	*h*°
Ba(Zn_0.85_Co_0.15_)_2_Si_2_O_7_	28.6	+52.2	−65.5	83.8	308.6
Co_3_(PO_4_)_2_^a^	46	+33	−32	44.3	315.9
NH_4_MnP_2_O_7_^a^	31	+39	−21	46.0	331.7

a Cited from ref. 20.

### Chemical stability tests

The chemical stability of the Ba(Zn_0.85_Co_0.15_)_2_Si_2_O_7_ pigment was also evaluated using a powder sample. The pigment was soaked into 4% acetic acid and 4% ammonium bicarbonate. After leaving them at room temperature for 2 h, the pigments were washed with deionized water and ethanol, and then dried at room temperature. The colour of the pigment after the leaching test was evaluated using the calorimeter. As seen in Table 5, the colour of the present Ba(Zn_0.85_Co_0.15_)_2_Si_2_O_7_ pigment was almost unchanged.

**Table 5:** The L*a*b*Ch° colour coordinates of the Ba(Zn_0.85_Co_0.15_)_2_Si_2_O_7_ pigment before and after the acid and base resistance tests

Pigment	L*	a*	b*	*C*	*h*°
Non-treatment	28.6	+52.2	−65.5	83.8	308.6
4% CH_3_COOH	28.0	+57.0	−69.4	89.8	309.4
4% NH_4_HCO_3_	25.7	+56.2	−67.8	88.1	309.7

## Conclusions

Ba(Zn_1−*x*_Co_*x*_)_2_Si_2_O_7_ (0 ≤ *x* ≤ 0.50) solid solutions were successfully synthesized as novel blue-violet inorganic pigments. The samples strongly absorbed the visible light from 550 to 650 nm (green to orange), which was originated by the d–d transition of tetrahedrally coordinated Co^2+^. Ba(Zn_0.85_Co_0.15_)_2_Si_2_O_7_ showed the most intense colour among the samples, and the L*a*b*Ch° parameters were L* = 28.6, a* = +52.2, b* = −65.5, *C* = 66.3, and *h*° = 308.6. The absolute values of a* and b* of Ba(Zn_0.85_Co_0.15_)_2_Si_2_O_7_ were significantly larger than those of the commercial Co_3_(PO_4_)_2_ (a* = +33 and b* = −32) and NH_4_MnP_2_O_7_ (a* = +39 and b* = −21) pigments. Furthermore, the Ba(Zn_0.85_Co_0.15_)_2_Si_2_O_7_ pigment has excellent chemical resistance and thermal stability. These results indicate that Ba(Zn_0.85_Co_0.15_)_2_Si_2_O_7_ could serve as an effective alternative to the conventional blue-violet inorganic pigments.

## Conflicts of interest

There are no conflicts to declare.

## Supplementary Material
